# Neoadjuvant chemotherapy versus primary debulking surgery in advanced epithelial ovarian cancer: A meta-analysis of peri-operative outcome

**DOI:** 10.1371/journal.pone.0186725

**Published:** 2017-10-23

**Authors:** Lijuan Yang, Bo Zhang, Guangyang Xing, Jingran Du, Bin Yang, Qianqian Yuan, Yongxiu Yang

**Affiliations:** 1 Department of Obstetrics and Gynecology, The First Affiliated Hospital of Lanzhou University, Lanzhou, Gansu, China; 2 Department of Orthopaedics, The Second Hospital of Lanzhou University, Lanzhou, Gansu, China; West China Second Hospital, Sichuan University, CHINA

## Abstract

**Objective:**

To assess whether neoadjuvant chemotherapy (NACT) is superior to primary debulking surgery (PDS) with regard to optimal cytoreduction, peri-operative morbidity, mortality, and quality of life (QOL) in advanced epithelial ovarian cancer (EOC).

**Methods:**

We searched the PubMed, Embase, Cochrane Central Register of Controlled Trials, Web of Science, Registers of Clinical Trials for randomized controlled trials (RCTs) comparing NACT to PDS in women with Federation of International Gynaecologists and Obstetricians stage Ⅲ-Ⅳ EOC. RevMan 5.3 software was utilized for statistical analysis.

**Results:**

Four RCTs involving 1,607 women with advanced EOC were included. Compared with PDS, NACT provided a higher rate of complete cytoreduction (risk ratio [RR], 1.95; 95% confidence interval [CI], 1.33 to 2.87), optimal cytoreduction (RR: 1.61 [95%CI: 1.05 to 2.47]), but there was no significant difference in residual disease 0–1 cm (p = 0.49). NACT was associated with lower peri-operative morbidity with respect to infection (RR: 0.30 [95% CI: 0.16 to 0.56]), gastrointestinal fistula (RR: 0.24 [95% CI: 0.06 to 0.95]), any grade 3 or 4 adverse event (RR: 0.29 [95% CI: 0.11 to 0.78]), and less post-surgical death within 28 days (RR: 0.14 [95% CI: 0.04 to 0.49]). NACT provided better QOL in terms of fatigue (weight mean difference [WMD], -3.28; [95% CI: -3.99 to -2.57]), role functioning (WMD: 5.29 [95% CI: 4.44 to 6.14]), emotional functioning (WMD: 6.19 [95% CI: 5.57 to 6.82]), and cognitive functioning (WMD: 1.02 [95% CI: 0.43 to 1.61]) at 6-month follow-up compared with PDS.

**Conclusions:**

NACT is associated with superior optimal cytoreduction, lower peri-operative morbidity as well as post-surgical mortality, and better QOL compared to initial surgery in patients with advanced EOC. Future research should focus on improving the efficacy of NACT.

## Introduction

Epithelial ovarian cancer (EOC) is the leading cause of death from gynecologic malignancy and is the seventh most common cancer among women worldwide [1]. It is estimated that over 80% of cases are diagnosed at metastatic, Federation of International Gynaecologists and Obstetricians (FIGO) stage Ⅲ and Ⅳ diseases at the time of clinical presentation [2]. As such, the 5-year survival rate for women with advanced EOC is approximately 30% [3]. Primary debulking surgery (PDS) followed by platinum-based adjuvant chemotherapy is considered standard of care. PDS is conducted to achieve optimal cytoreduction (residual disease ≤ 1 cm [4], ideally to achieve no visible disease) as the extent of tumour cytoreduction is considered the most important prognostic factors for survival of advanced EOC [5]. Nonetheless, PDS is not the first-line treatment for women with unresectable disease, and PDS is associated with increased peri-operative morbidity, mortality, and diminished quality of life (QOL) as an extensive debulking procedure [6, 7]. Given the disadvantages of PDS, alternative treatment strategies may be beneficial.

Neoadjuvant chemotherapy (NACT), defined as the administration of platinum-based chemotherapy prior to interval debulking surgery (IDS) to reduce tumour size, is considered an alternative to PDS for women with advanced EOC [7, 8]. Compared with aggressive PDS, recent researches have suggested that NACT is associated with a higher rate of optimal cytoreduction for patients with advanced EOC [6, 9, 10], and that the use of NACT may increase the proportion of patients with low peri-operative morbidity and good QOL [8–12]. However, it is confirmed by the high quality RCTs that NACT does not improve overall survival (OS) and progression-free survival (PFS) compared to PDS [7, 8]. Currently, guidelines from the Society for Gynecologic Oncology (SGO), and the American Society for Clinical Oncology (ASCO), have recommended NACT for patients with high perioperative risk, or low likelihood of achieving optimal debulking [13].

NACT with aforementioned advantages for the treatment of advanced EOC has remained controversial, despite substantial studies suggesting that NACT is superior to primary surgery [6, 9–11]. Three retrospective or non-randomized trials including women with bulky stage ⅢC or Ⅳ EOC concluded that NACT provided a higher rate of optimal cytoreduction and lower peri-operative morbidity compared with PDS [6, 10, 11]. In contrast, the results of several retrospective or non-randomized trials showed that women with stages ⅢC and Ⅳ EOC allocated to NACT had lower optimal cytoreduction rate [14–16], or similar optimal debulking [17, 18] and peri-operative morbidity [14, 19, 20]. Furthermore, even among a few randomized controlled trials (RCTs) [7, 8, 12, 21, 22] which assessed the outcomes of NACT in women with advanced EOC, these are conflicting conclusions with regard to optimal cytoreduction and QOL. Four recently published RCTs demonstrated that NACT was associated with a significant improvement in optimal cytoreduction [7, 8, 22] and QOL [12], while several RCTs showed similar optimal debulking [12] and QOL [21] for patients with advanced EOC between NACT and primary surgery.

A few meta-analytical studies reviewed the role of NACT for women with stage Ⅲ or Ⅳ EOC. Three meta-analysis including only RCTs reported that NACT provided similar benefits of OS and PFS compared to PDS for women with advanced EOC [23–25]. Zeng et al. [25] demonstrated that NACT was associated with a higher rate of optimal debulking in women with advanced EOC compared with PDS, which was based on the pooled estimate of two included RCTs [7, 8]. Besides, the meta-analysis performed by Morrison and colleagues [24] included one RCT and indicated that significant difference occurred between NACT and PDS group with respect to some surgically related serious adverse effects in patients with advanced EOC. To our knowledge, there was no meta-analysis systematically comparing NACT with PDS with respect to peri-operative morbidity, mortality, and QOL for women with stage Ⅲ or Ⅳ EOC.

Hence, the aim of our systematic reviews and meta-analyses focusing only on high quality trials was to assess whether NACT is superior to PDS with regard to optimal cytoreduction as well as peri-operative morbidity, mortality, and QOL in advanced EOC.

## Materials and methods

### Article search

We followed the Preferred Reporting Items for Systematic Reviews and Meta-Analyses (PRISMA) guidelines (S1 File) [26]. The PubMed, Embase, Cochrane Central Register of Controlled Trials, Web of Science, and Registers of Clinical Trials were searched for relevant studies from database inception until March, 2017. No language restriction was imposed. The search terms (Table 1) included “Ovarian Neoplasms”, “Ovarian Carcinoma”, “Ovarian cancer”, “Chemotherapy, Adjuvant”, “neoadjuvant chemotherapy”, “Cytoreduction Surgical Procedures”, “debulking surgery”, and “cytoreductive surgery”. Moreover, the reference lists of the relevant literatures were also screened.

**Table 1:** Search strategy.

Ovarian Neoplasms		Neoadjuvant chemotherapy		Cytoreduction Surgical
OR		OR		OR
● Ovarian Carcinoma	**AND**	● NACT ● Adjuvant Chemotherapy ● Adjuvant Drug Therapy	**AND**	● debulking surgery ● cytoreduct* surgical ● cytoreductive surgery
● Ovarian Cancer				
● Sertoli-Leydig Cell Tumor				
● Thecoma				
● Brenner Tumor				
● Ovar* Neoplasm*				

### Eligibility criteria

The studies fulfilled the following predefined criteria: 1) subjects were patients pathologically diagnosed with FIGO stage Ⅲ or Ⅳ EOC; 2) interventions were platinum-based NACT followed by IDS and chemotherapy or PDS followed by platinum-based chemotherapy, compared with PDS followed by NACT then IDS followed by chemotherapy; and 3) published RCTs. Studies were excluded if they were review literature or conference abstracts, ongoing studies, or they assigned patients received PDS followed by NACT then IDS followed by chemotherapy to NACT arm.

### Data extraction

Two authors (Yang and Zhang) independently applied the inclusion criteria to the identified studies. A final agreement on inclusion was reached by consensus after discussion. Two authors (Xing and Zhang) extracted characteristics of the included articles independently. Study quality was assessed by two reviewers (Yuan and Du) using the Cochrane Collaboration’s risk of bias tool for RCT.

### Meta-analysis

The relative risks (RRs) and weight mean differences (WMDs) were used to report pooled estimates as our measure of the effect based on 95% confidence intervals (CIs). We chose to use I^2^ statistic to measure statistical heterogeneity [27]. An I^2^ ≤ 25%, 26% to 50%, and > 50% were considered low, moderate, and high heterogeneity, respectively [28]. Fixed effects model was used when I^2^ ≤ 25% [27]. Otherwise, random-effects model was presented. The presence of publication bias was assessed via Begg's funnel plots and Egger's linear regression tests for the primary outcomes [29]. Sensitivity analysis was conducted to investigate the causes of heterogeneity [30]. The meta-analysis was performed using RevMan 5.3 software from the Cochrane reviews.

## Results

### Identification of included studies

**Fig 1:**
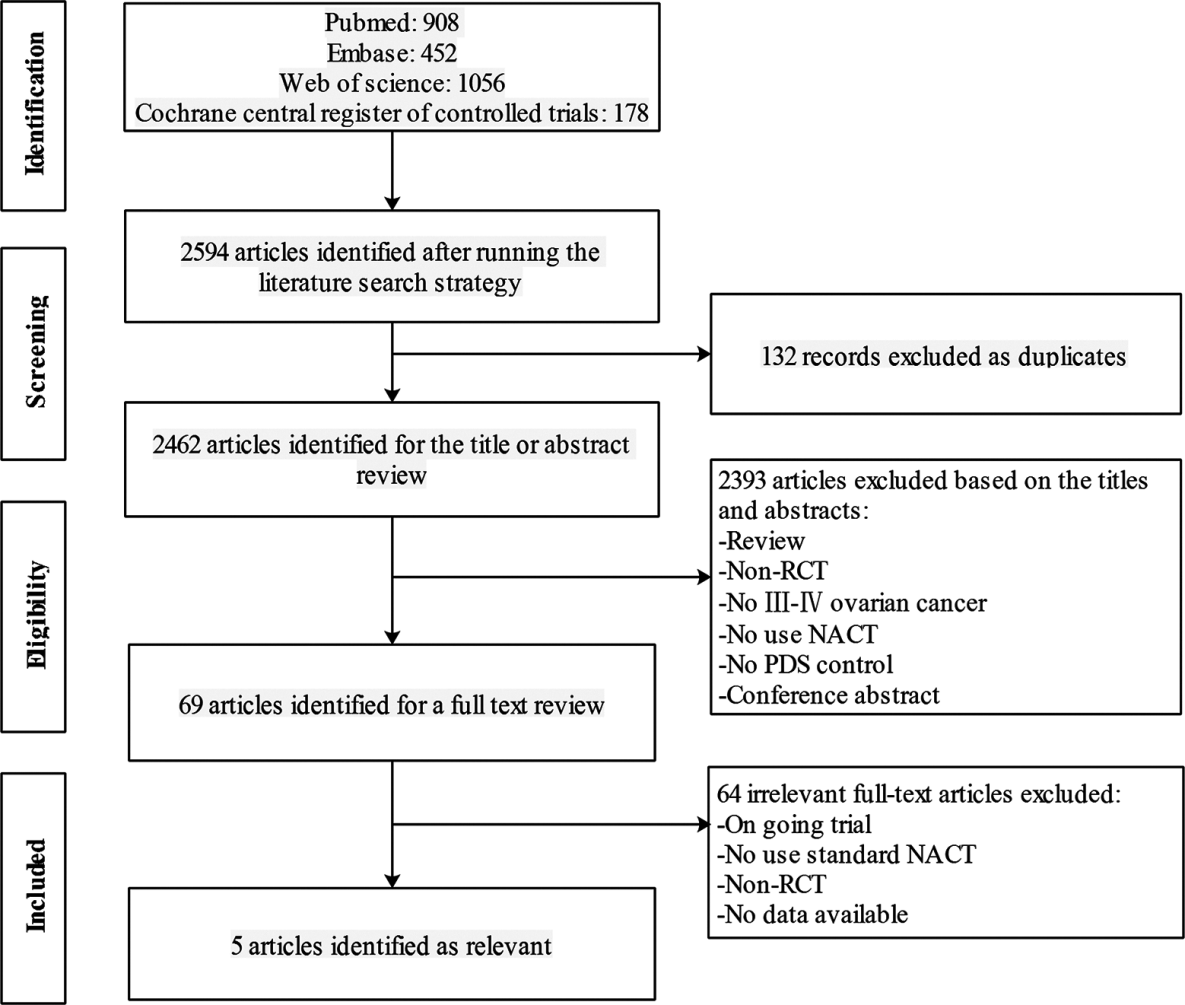
Flowchart of literature search. RCT, randomized controlled trial. NACT, neoadjuvant chemotherapy followed by interval debulking surgery. PDS, primary cytoreductive surgery.

A total of 2,594 articles were yielded after electronic literature search (Fig 1). Of these, 132 articles were excluded as duplicate publications and 2,393 were excluded after screening of the title and/or abstract. After a full-text review, 64 were excluded from the remaining 69 articles. Finally, the meta-analysis included 4 RCTs [7, 8, 12, 21, 22]. Among the five included articles [7, 8, 12, 21, 22], Vergote et al. [7] and Greimel et al. [21] were the main endpoint and secondary endpoint of the EORTC 55971 trial.

### Studies characteristics and quality assessment

**Fig 2:**
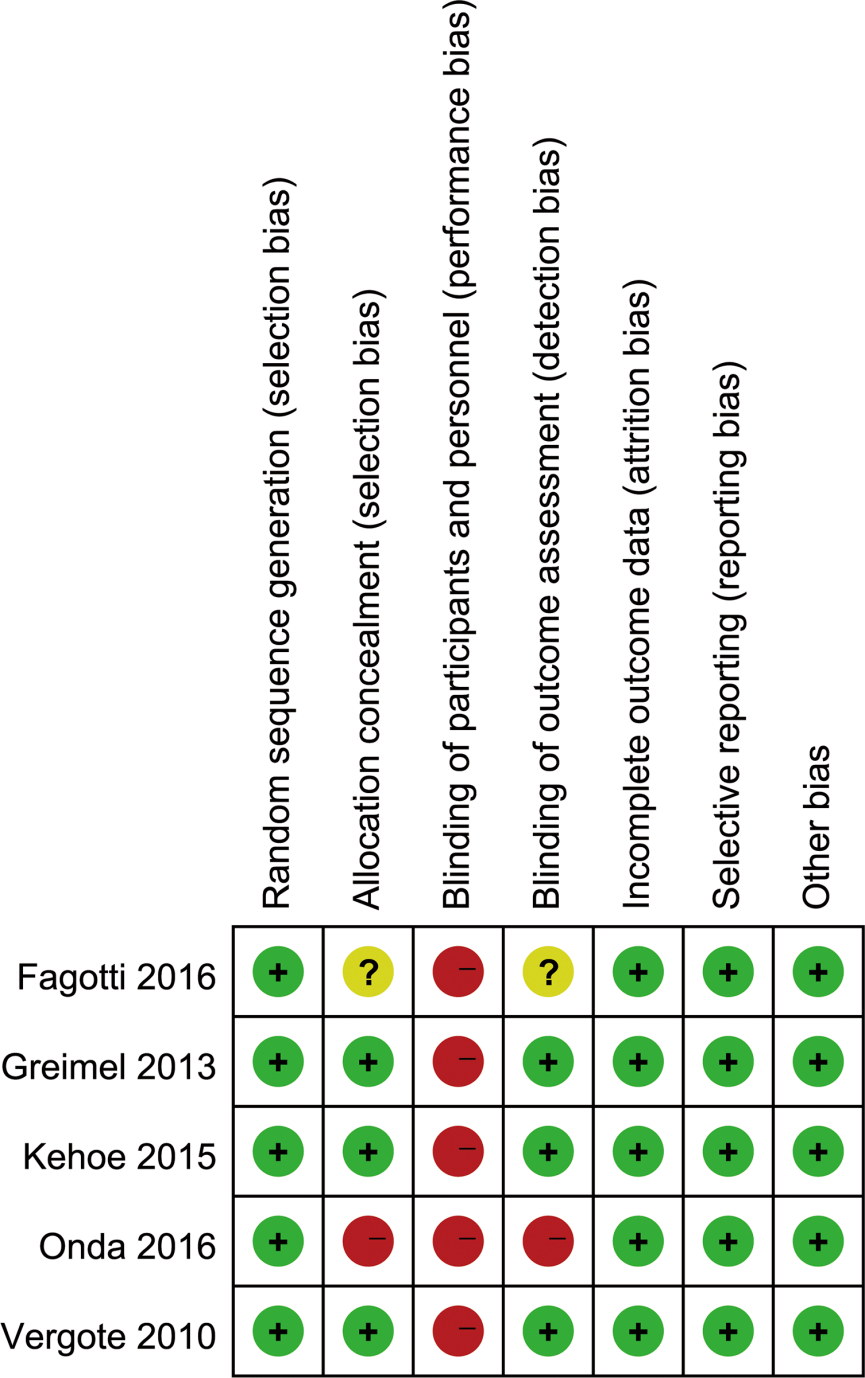
The quality assessment for selected randomized controlled trials. Low risk of bias (green circles), unclear risk of bias (yellow circles) and high risk of bias (red circles).

4 RCTs [7, 8, 12, 21, 22] were included in the meta-analysis due to Vergote et al. [7] and Greimel et al. [21] belong to the same RCT. Characteristics of included articles are presented in Table 2. A total of 1,607 women with stage Ⅲ-Ⅳ EOC undergoing NACT or PDS were included. The quality assessment for selected studies is described in Fig 2. We only included RCTs thus minimizing the risk of bias within articles.

**Table 2:** Details of included studies.

Study^a^	No. Patients (NACT/PDS)	Age(Year)^b^ NACT/PDS	FIGO Stage N (%)		Intervention		Follow-up(Years)
			NACT	PDS	NACT	PDS	
Greimel *et al*. 2013, Austria	203/201	63(33–81)/62(25–86)	Ⅲc	Ⅲc	NACT×3cycles+IDS+Chemotherapy×3cycles	PDS+Chemotherapy×6cycles	4.7
			253(75.7%)	257(76.5%)			
			Ⅳ	Ⅳ			
			81(24.3%)	77(22.9%)			
Vergote *et al*. 2010, Belgium	334/336	63(33–81)/62(25–86)	Ⅲc	Ⅲc	NACT×3cycles+IDS+Chemotherapy×3cycles	PDS+Chemotherapy×6cycles	4
			253(75.7%)	257(76.5%)			
			Ⅳ	Ⅳ			
			81(24.3%)	77(22.9%)			
Kehoe *et al*. 2015, England	274/276	65(34–88)/66(26–87)	Ⅲ	Ⅲ	NACT×3cycles+IDS+Chemotherapy×3cycles	PDS+Chemotherapy×6cycles	4.4
			206(75%)	206(75%)			
			Ⅳ	Ⅳ			
			68(25%)	70(25%)			
Onda *et al*. 2016, Japan	130/147	60.5(36–75)/59(30–75)	Ⅲ	Ⅲ	NACT×4cycles+IDS+Chemotherapy×4cycles	PDS+Chemotherapy×8cycles	5
			105(69.1%)	100(67.1%)			
			Ⅳ	Ⅳ			
			47(30.9%)	49(32.9%)			
Fagotti *et al*. 2016, Italy	55/55	55(36–75)/54(39–74)	Ⅲc	Ⅲc	NACT×3cycles+IDS+Chemotherapy×3cycles	PDS+Chemotherapy×6cycles	5
			51(92.7%)	47(85.5%)			
			Ⅳ	Ⅳ			
			4(7.3%)	8(14.5%)			

NACT, neoadjuvant chemotherapy; IDS, interval debulking surgery; PDS, primary cytoreductive surgery; FIGO, Federation of International Gynaecologists and Obstetricians.

^a^Study, Vergote et al. [7] and Greimel et al. [21] were belong to the same RCT

^b^Age, median (minimum age-maximum age).

### Postoperative complications and mortality

**Fig 3:**
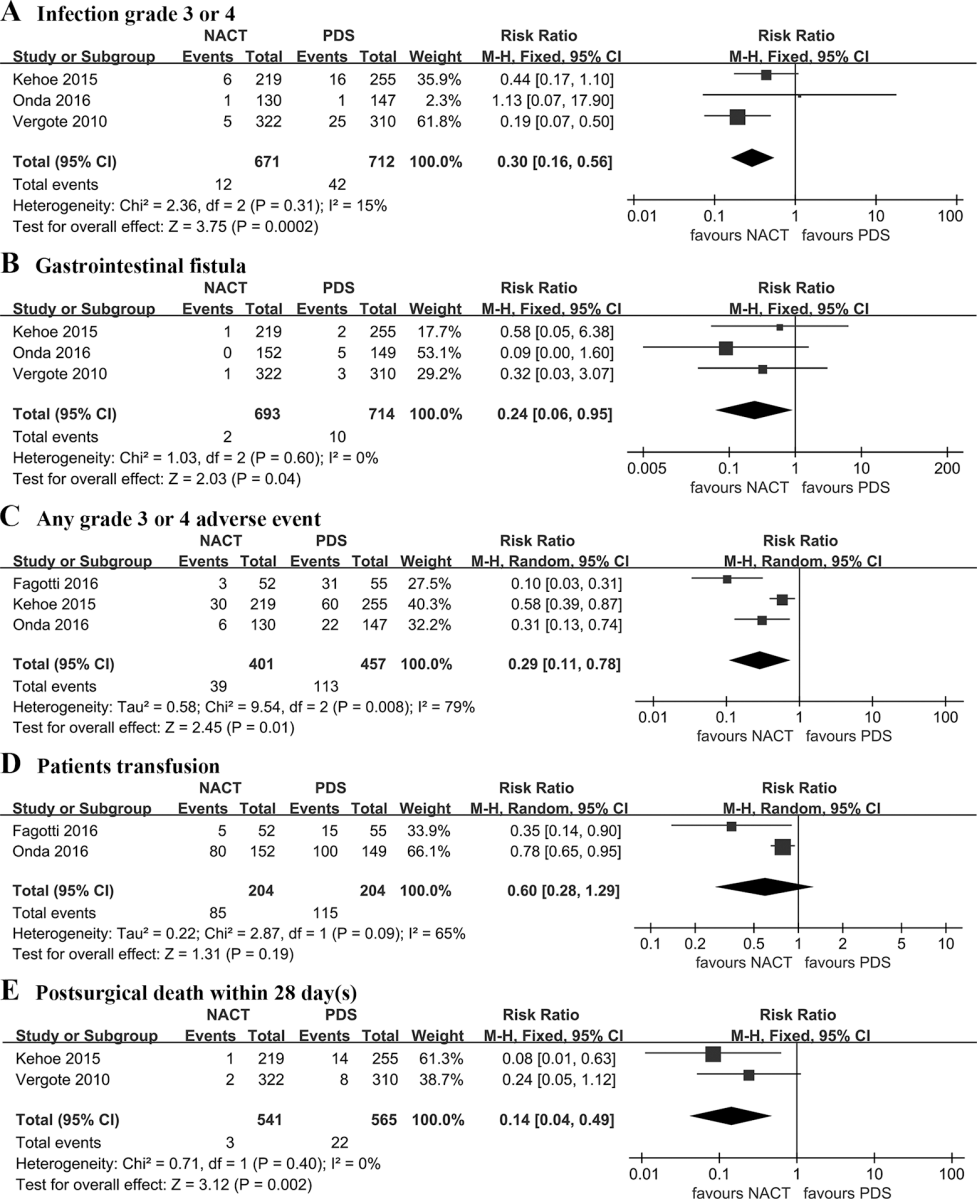
Post-operative complications and mortality. NACT: neoadjuvant chemotherapy followed by interval debulking surgery. PDS: primary cytoreductive surgery.

Pooled analysis showed that NACT significantly reduced the risk of post-operative complications with regard to infection grade 3 or 4 (RR: 0.30 [95% CI, 0.16 to 0.56], p = 0.0002, I^2^ = 15%), gastrointestinal fistula (RR: 0.24 [95% CI, 0.06 to 0.95], p = 0.04, I^2^ = 0%), any grade 3 or 4 adverse event (RR: 0.29 [95% CI, 0.11 to 0.78], p = 0.01, I^2^ = 0%), postsurgical death within 28 days (RR: 0.14 [95% CI, 0.04 to 0.49], p = 0.002, I^2^ = 0%). However, patients transfusion was similar between NACT and PDS group (RR: 0.60 [95% CI, 0.28 to 1.29], p = 0.19, I^2^ = 65%) (Fig 3).

### Quality of life

**Fig 4:**
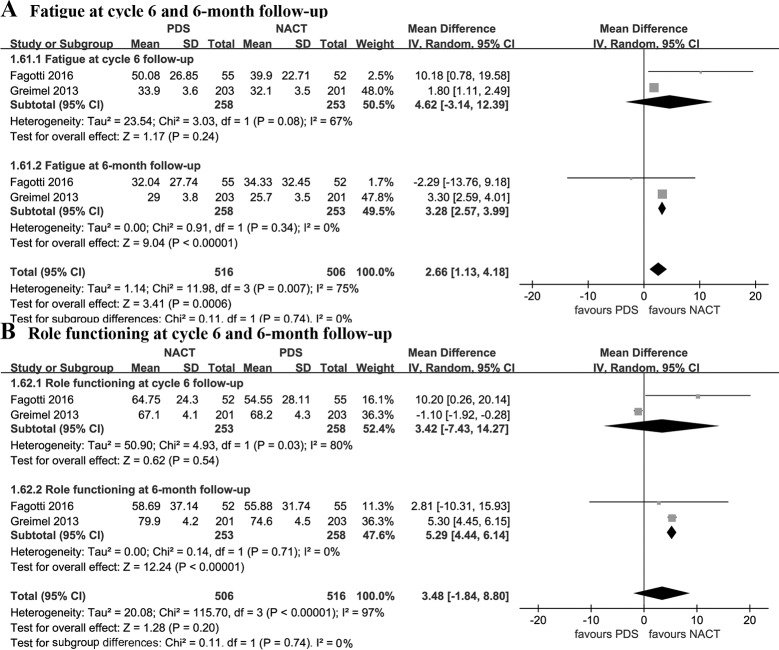
Fatigue and role functioning at cycle 6 and 6-month follow-up. NACT, neoadjuvant chemotherapy followed by interval debulking surgery; PDS, primary debulking surgery.

**Fig 5:**
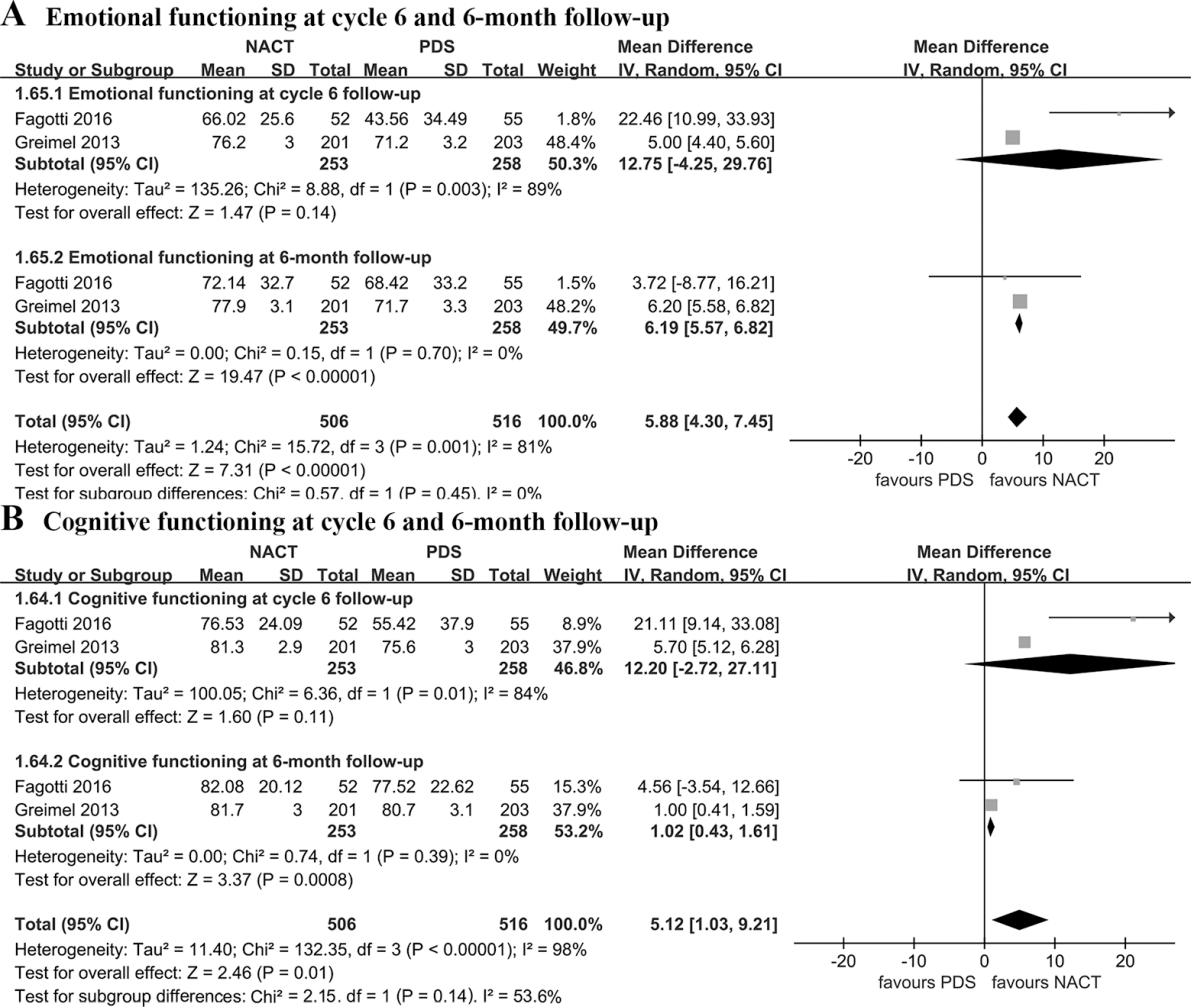
Emotional functioning and cognitive functioning at cycle 6 and 6-month follow-up. NACT, neoadjuvant chemotherapy followed by interval debulking surgery; PDS, primary debulking surgery.

Pooled estimates demonstrated that NACT was associated with better QOL in terms of fatigue (WMD: -3.28 [95% CI, -3.99 to -2.57], p < 0.00001, I^2^ = 0%), role functioning (WMD = 5.29 [95% CI, 4.44 to 6.14], p < 0.00001, I^2^ = 0%), emotional functioning (WMD: 6.19 [95% CI, 5.57 to 6.82], p < 0.00001, I^2^ = 0%), and cognitive functioning (WMD = 1.02 [95% CI, 0.43 to 1.61], p = 0.0008, I^2^ = 0%) at 6-month follow-up. Nonetheless, there was no significant difference between the two groups with regard to fatigue, role functioning, emotional functioning, cognitive functioning at cycle 6 follow-up (p > 0.05) (Figs 4 and 5).

### Extent of residual disease

**Fig 6:**
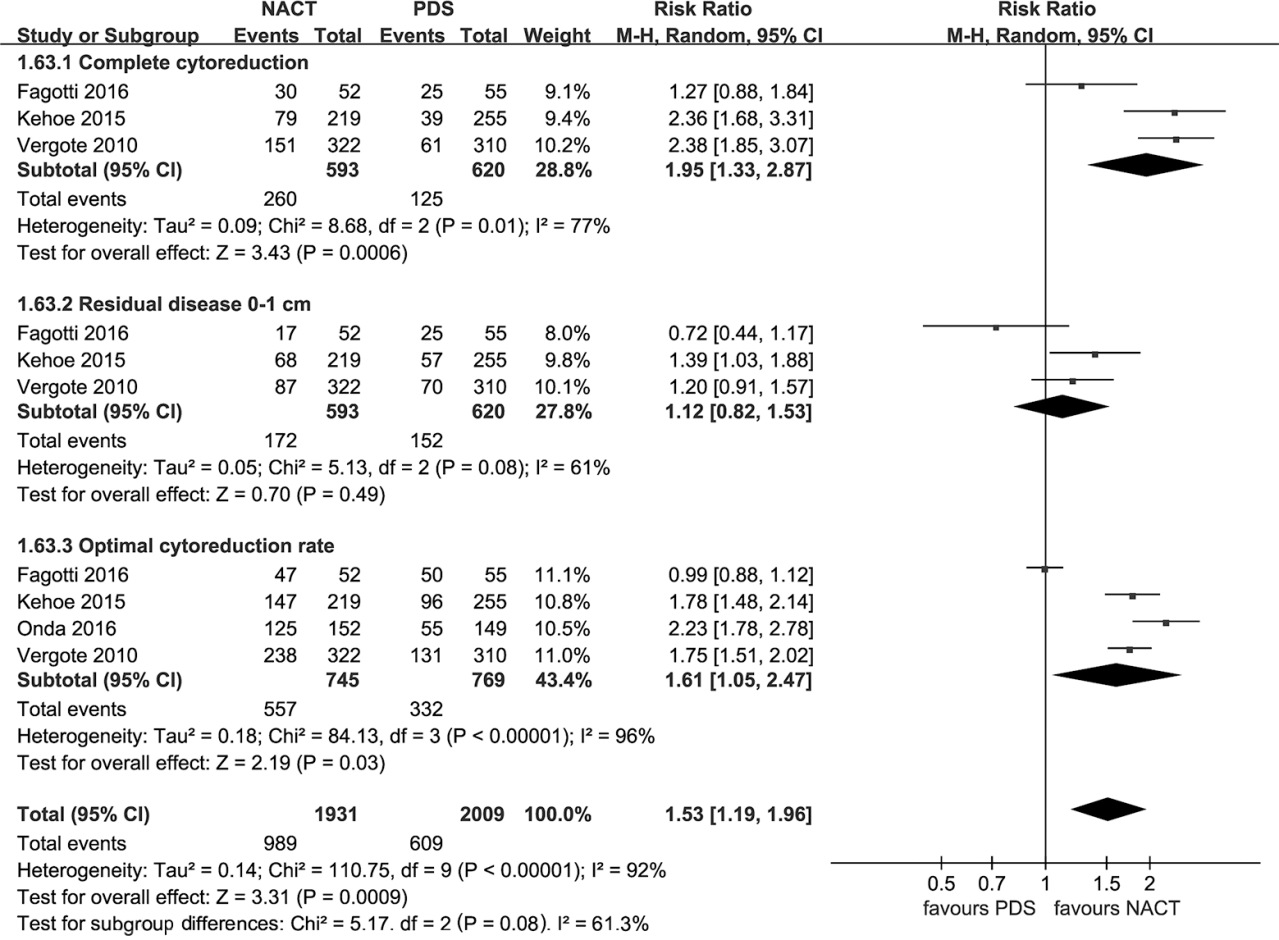
Extent of residual disease. NACT: neoadjuvant chemotherapy followed by interval debulking surgery. PDS: primary debulking surgery.

Use of a random-effects model indicated that NACT provided a higher rate of complete cytoreduction (RR: 1.95 [95%CI, 1.33 to 2.87]; p = 0.0006; I^2^ = 77%), optimal cytoreduction (RR: 1.61 [95% CI, 1.05 to 2.47], p = 0.01, I^2^ = 96%), but the rate of residual disease 0–1 cm was similar between the two groups (p = 0.49) (Fig 6).

### Publication bias and sensitivity analysis

As summarized in S1 Fig, Begg’s funnel plots for the primary results did not show obvious asymmetry. Similarly, Egger's linear regression tests suggested no significant publication bias (all p > 0.1). Inconsistency across trials was high for the results of any grade 3 or 4 adverse event (I^2^ = 79%), complete cytoreduction (I^2^ = 77%), residual disease 0–1 cm (I^2^ = 61%), and optimal cytoreduction (I^2^ = 96%). Fagotti et al. [12] was found to probably contribute to the high heterogeneity in the sensitivity analysis. After removing this trial, the pooled estimates remained statistically significant difference between NACT and PDS group in terms of any grade 3 or 4 adverse event (RR: 0.48 [95% CI, 0.27 to 0.85], p = 0.01, I^2^ = 41%), complete cytoreduction (RR: 2.37 [95% CI, 1.94 to 2.91], p < 0.00001, I^2^ = 0%), residual disease 0–1 cm (RR: 1.28 [95% CI, 1.04 to 1.57], p = 0.02, I^2^ = 0%). After excluding Fagotti et al. [12], the pooled estimate of optimal cytoreduction rate remained significantly statistical difference between the two groups (RR: 1.87 [95% CI: 1.63 to 2.14], p < 0.00001, I^2^ = 41%).

## Discussion

The meta-analysis of 4 RCTs, involving 1,607 women with stage Ⅲ or Ⅳ EOC, was conducted to systematically assess peri-operative outcomes with respect to optimal cytoreduction, peri-operative morbidity, mortality, and QOL in advanced EOC. Our results demonstrated that NACT provided a higher rate of complete cytoreduction and optimal cytoreduction than PDS. Furthermore, NACT was associated with lower post-operative complications (grade 3 or 4) with regard to infection, gastrointestinal fistula, any adverse event, and less post-surgical death within 28 days. NACT provided better QOL in terms of fatigue, role functioning, emotional functioning, and cognitive functioning at 6-month follow-up compared with PDS, but there was no significant difference in that at cycle 6 follow-up between NACT and PDS group.

Recently, guidelines from the SGO and ASCO have reached similar conclusions to our study based on 4 RCTs, but two of these trails were available only as oral presentations from national meetings [13]. The published guidelines suggested that for selected patients with stage ⅢC or Ⅳ EOC, NACT is associated with a higher rate of complete cytoreduction, less perioperative complications and mortality [13]. After further research, our article including 4 published RCTs demonstrated that NACT provided better QOL, lower post-operative complications (grade 3 or 4) with regard to infection, gastrointestinal fistula, any adverse event, and less post-surgical death within 28 days. Besides, NACT was associated with a higher rate of complete cytoreduction than PDS, however, there was no significant difference in residual disease 0–1 cm between the two groups. Consequently, our results may provide useful information for the treatment of stage Ⅲ or Ⅳ EOC.

This meta-analysis demonstrated that NACT was associated with a higher rate of optimal cytoreduction compared with PDS in advanced EOC. The random effects model indicated substantial heterogeneity among the included trials. Fagotti et al. [12] was the cause of high heterogeneity of the results with regard to optimal cytoreduction. Fewer women with stage Ⅳ EOC (11%) were included in the study performed by Fagotti et al. [12] which reported that the optimal debulking rate was similar between NACT and primary surgery. This is in contrast to the other two studies conducted by Vergote [7] and Kehoe [8] in which about 25% women with stage Ⅳ EOC were included, and the results of the two RCTs [7, 8] demonstrated that NACT provided a higher rate of optimal cytoreduction compared with PDS. Given the complex nature of advanced EOC, many gynecologic oncologists have now proposed NACT for the treatment of stage Ⅳ EOC [31]. More specifically, women with stage Ⅳ disease and metastatic tumors > 45 mm benefited more from NACT [32]. Besides, the pooled estimates in two included RCTs [7, 8] demonstrated that the RR of complete cytoreduction was higher than that of residual disease 0–1 cm (2.37 versus 1.28), suggesting that the rate of complete cytoreduction in NACT was more remarkable. Similarly, some investigators have a strong believe that complete cytoreduction is easier to attain after NACT, because small metastatic nodules, such as generally occur on the bowel or its mesentery and diaphragm, will usually disappear macroscopically if the disease is chemo-sensitive [33]. Hence, we suggest that complete cytoreduction should be the goal of NACT followed by IDS.

The included RCTs reported complete cytoreduction rates of 39–58% in NACT or 17–45% in PDS [7, 8, 12], which were overall lower than that seen in several retrospective studies [34–36]. Several factors influencing the complete cytoreduction rate are based on individual perceptions regarding disease status, age, performance status, and co-morbidities [37]. However, the main factor is attributed to the purposeful selection of the cohort with bulky initial disease in the included RCTs [7, 8, 12, 21]. 40% of patients in the EORTC trial had metastatic lesions >10 cm [7]. 73% of the recruited patients in the CHORUS trial had metastatic lesions >5 cm [8]. Likewise, there is bias in population selection of a cohort with high tumour load in the study by Fagotti et al. [12]. In other words, a majority of the recruited patients in these RCTs were deemed unresectable at primary surgery. Hence, their results should be treated with caution. Furthermore, the blind extrapolation of NACT to all patients with advanced EOC risks that a significant proportion of patients will be withheld from potentially more curative treatment. Hitherto, many gynecologic oncologists have suggested that the majority of patients with stage Ⅳ EOC should be treated with NACT, whereas in stage ⅢC patients who can achieve optimal cytoreduction should be performed with PDS [32, 38–40].

Our results indicated that NACT was associated with significantly lower peri-operative complications (grade 3 or 4) with respect to infection, gastrointestinal fistula, any adverse event, and less post-surgical death within 28 days, which are consistent with the previous meta-analysis based on one RCT [24]. The postsurgical mortality in the NACT group was less than 1%, whereas 4% of women with advanced EOC in the PDS group died within 28 days after surgery. This result lies within the range of peri-operative morbidities and mortalities that reported in the literature from 15% to 45% [15]. These findings may suggest that IDS following NACT is a less invasive surgery compared to primary surgery. The aggressive upper abdominal procedures are usually performed in PDS for patients with advanced EOC to achieve a longer median survival, thus increase the morbidity and mortality in those patients [6]. Generally, complications of PDS include infection, bleeding, gastrointestinal injury, or vascular injury [41]. It is estimated that the occurrence of more than two post-operative complications after debulking surgery was associated with a significantly decreased survival for women with advanced EOC [33]. Hence, NACT followed by IDS can be considered an alternative first-line treatment for women with advanced EOC in whom primary surgery is contraindicated due to co-morbidity [7, 42].

This meta-analysis reported that NACT provided better QOL in terms of fatigue, role functioning, emotional functioning, and cognitive functioning at 6-month follow-up compared with PDS, but there was no significantly statistical difference with regard to that at cycle 6 follow-up between the two groups. Besides, several QOL scores in the two included RCTs [12, 21] showed an improvement over time in both the NACT and PDS group after initiating treatment. Our results are consistent with the RCT performed by Kehoe et al. [8], which indicated that NACT was associated with superior QOL at 6- and 12-month follow-up. The worse QOL in PDS group may be attributed to the significantly high post-operative complications due to the extensive primary surgery. Similarly, some investigators reported that women with advanced EOC frequently suffered a variety of treatment-related side effects which may diminish their QOL [43]. Nonetheless, one of the included studies performed by Greimel et al. [21], which selected patients with a higher optimal debulking rate and better survival outcomes among the selected institutions with good QOL compliance, demonstrated that NACT was associated with similar QOL compared with PDS. Given the small number of included studies when pooled estimates of QOL in our meta-analysis, we hypothesized that further research probably has a significant impact on our confidence in the assessments of QOL and may change the assessments.

Despite that patients allocated to NACT showed statistically and clinically improved outcomes compared to PDS patients with respect to optimal cytoreduction, peri-operative morbidity, mortality, and QOL, NACT failed to improve OS and PFS in women with advanced EOC [7, 8]. Among the benefits of NACT, optimal cytoreduction is considered the most important prognostic factors for survival of advanced EOC [5]. Besides, An early GOG study showed that patients with initially large-volume disease had worse outcomes than patients with initially small-volume disease after both groups were optimally debulking, calling into question prognostic factors other than optimal cytoreduction as being important in predicting survival, such as the tumor burden [44]. Hence, it might contribute to the similar OS and PFS between NACT and PDS arm that the EORTC 55971 and CHORUS trials extensively enrolled patients with bulky initial disease in both groups.

Although we only included RCTs in this meta-analysis in order to minimize the risk of bias, there remain potential limitations to this study which must be considered in interpreting data. The most significant limitation of this study is the heterogeneity which attributed to different study designs, surgical procedures, and chemotherapy regimens among the included studies. We used a random-effects model to mitigate the underlying effect of high heterogeneity on the results and subsequently explored the causes of heterogeneity using sensitivity analysis.

In summary, NACT is associated with superior optimal cytoreduction, lower peri-operative morbidity as well as post-surgical mortality, and improved QOL compared with initial surgery, suggesting that NACT remains an attractive treatment regimen in patients with advanced EOC. Future research should focus on improving the effectiveness of NACT.

## Supporting information

S1 File PRISMA 2009 checklist. (DOC)

S1 Fig Begg’s funnel plots for primary outcomes in the meta-analysis. A, infection grade 3 or 4; B, gastrointestinal fistula; C, any grade 3 or 4 adverse event; D, patients transfusion; E, postsurgical death within 28 days; F, complete cytoreduction; G, residual disease 0–1 cm; H, optimal cytoreduction rate. RR = risk ratio, SE = standard error.
(TIF)

## References

[1] Siegel RL, Fedewa SA, Miller KD, Goding-Sauer A, Pinheiro PS, Martinez-Tyson D, et al Cancer statistics for Hispanics/Latinos, 2015. CA: a cancer journal for clinicians. 2015;65: 457–80. doi: 10.3322/caac.21314 26375877 10.3322/caac.21314

[2] Minig L, Zorrero C, Iserte PP, Poveda A. Selecting the best strategy of treatment in newly diagnosed advanced-stage ovarian cancer patients. World journal of methodology. 2015;5: 196–202. doi: 10.5662/wjm.v5.i4.196 26713279 10.5662/wjm.v5.i4.196 PMC4686416

[3] Seidman JD, Yemelyanova A, Cosin JA, Smith A, Kurman RJ. Survival rates for international federation of gynecology and obstetrics stage III ovarian carcinoma by cell type: a study of 262 unselected patients with uniform pathologic review. International journal of gynecological cancer: official journal of the International Gynecological Cancer Society. 2012;22: 367–71. doi: 10.1097/IGC.0b013e31823c6f80 22237384 10.1097/IGC.0b013e31823c6f80

[4] Fader AN, Rose PG. Role of surgery in ovarian carcinoma. Journal of clinical oncology: official journal of the American Society of Clinical Oncology. 2007;25: 2873–83. doi: 10.1200/jco.2007.11.0932 17617518 10.1200/JCO.2007.11.0932

[5] Stuart GC, Kitchener H, Bacon M, duBois A, Friedlander M, Ledermann J, et al 2010 Gynecologic Cancer InterGroup (GCIG) consensus statement on clinical trials in ovarian cancer: report from the Fourth Ovarian Cancer Consensus Conference. International journal of gynecological cancer: official journal of the International Gynecological Cancer Society. 2011;21: 750–5. doi: 10.1097/IGC.0b013e31821b2568 21543936 10.1097/IGC.0b013e31821b2568

[6] Hou JY, Kelly MG, Yu H, McAlpine JN, Azodi M, Rutherford TJ, et al Neoadjuvant chemotherapy lessens surgical morbidity in advanced ovarian cancer and leads to improved survival in stage IV disease. Gynecologic oncology. 2007;105: 211–7. doi: 10.1016/j.ygyno.2006.11.025 17239941 10.1016/j.ygyno.2006.11.025

[7] Vergote I, Trope CG, Amant F, Kristensen GB, Ehlen T, Johnson N, et al Neoadjuvant chemotherapy or primary surgery in stage IIIC or IV ovarian cancer. The New England journal of medicine. 2010;363: 943–53. doi: 10.1056/NEJMoa0908806 20818904 10.1056/NEJMoa0908806

[8] Kehoe S, Hook J, Nankivell M, Jayson GC, Kitchener H, Lopes T, et al Primary chemotherapy versus primary surgery for newly diagnosed advanced ovarian cancer (CHORUS): an open-label, randomised, controlled, non-inferiority trial. Lancet (London, England). 2015;386: 249–57. doi: 10.1016/s0140-6736(14)62223-6 10.1016/S0140-6736(14)62223-6 26002111

[9] Fago-Olsen CL, Ottesen B, Kehlet H, Antonsen SL, Christensen IJ, Markauskas A, et al Does neoadjuvant chemotherapy impair long-term survival for ovarian cancer patients? A nationwide Danish study. Gynecologic oncology. 2014;132: 292–8. doi: 10.1016/j.ygyno.2013.11.035 24321400 10.1016/j.ygyno.2013.11.035

[10] Lee SJ, Kim BG, Lee JW, Park CS, Lee JH, Bae DS. Preliminary results of neoadjuvant chemotherapy with paclitaxel and cisplatin in patients with advanced epithelial ovarian cancer who are inadequate for optimum primary surgery. The journal of obstetrics and gynaecology research. 2006;32: 99–106. doi: 10.1111/j.1447-0756.2006.00359.x 16445534 10.1111/j.1447-0756.2006.00359.x

[11] Zheng H, Gao YN. Primary debulking surgery or neoadjuvant chemotherapy followed by interval debulking surgery for patients with advanced ovarian cancer. Chinese journal of cancer research = Chung-kuo yen cheng yen chiu. 2012;24: 304–9. doi: 10.3978/j.issn.1000-9604.2012.09.02 23358672 10.3978/j.issn.1000-9604.2012.09.02 PMC3551321

[12] Fagotti A, Ferrandina G, Vizzielli G, Fanfani F, Gallotta V, Chiantera V, et al Phase III randomised clinical trial comparing primary surgery versus neoadjuvant chemotherapy in advanced epithelial ovarian cancer with high tumour load (SCORPION trial): Final analysis of peri-operative outcome. European journal of cancer (Oxford, England: 1990). 2016;59: 22–33. doi: 10.1016/j.ejca.2016.01.017 26998845 10.1016/j.ejca.2016.01.017

[13] Wright AA, Bohlke K, Armstrong DK, Bookman MA, Cliby WA, Coleman RL, et al Neoadjuvant chemotherapy for newly diagnosed, advanced ovarian cancer: Society of Gynecologic Oncology and American Society of Clinical Oncology Clinical Practice Guideline. Gynecologic oncology. 2016;143: 3–15. doi: 10.1016/j.ygyno.2016.05.022 27650684 10.1016/j.ygyno.2016.05.022 PMC5413203

[14] Revaux A, Rouzier R, Ballester M, Selle F, Darai E, Chereau E. Comparison of morbidity and survival between primary and interval cytoreductive surgery in patients after modified posterior pelvic exenteration for advanced ovarian cancer. International journal of gynecological cancer: official journal of the International Gynecological Cancer Society. 2012;22: 1349–54. doi: 10.1097/IGC.0b013e318265d358 22954783 10.1097/IGC.0b013e318265d358

[15] Morice P, Dubernard G, Rey A, Atallah D, Pautier P, Pomel C, et al Results of interval debulking surgery compared with primary debulking surgery in advanced stage ovarian cancer. Journal of the American College of Surgeons. 2003;197: 955–63. doi: 10.1016/j.jamcollsurg.2003.06.004 14644284 10.1016/j.jamcollsurg.2003.06.004

[16] Markauskas A, Mogensen O, dePont Christensen R, Jensen PT. Primary surgery or interval debulking for advanced epithelial ovarian cancer: does it matter? International journal of gynecological cancer: official journal of the International Gynecological Cancer Society. 2014;24: 1420–8. doi: 10.1097/igc.0000000000000241 25180461 10.1097/IGC.0000000000000241

[17] Siesto G, Cavina R, Romano F, Vitobello D. Primary Debulking Surgery Versus Neoadjuvant Chemotherapy in Advanced Epithelial Ovarian Cancer: A Propensity-matched Analysis. American journal of clinical oncology. 2016 doi: 10.1097/coc.0000000000000262 10.1097/COC.0000000000000262 26757434

[18] Giannopoulos T, Butler-Manuel S, Taylor A, Ngeh N, Thomas H. Clinical outcomes of neoadjuvant chemotherapy and primary debulking surgery in advanced ovarian carcinoma. European journal of gynaecological oncology. 2006;27: 25–8. 16550963

[19] Kuhn W, Rutke S, Spathe K, Schmalfeldt B, Florack G, von Hundelshausen B, et al Neoadjuvant chemotherapy followed by tumor debulking prolongs survival for patients with poor prognosis in International Federation of Gynecology and Obstetrics Stage IIIC ovarian carcinoma. Cancer. 2001;92: 2585–91. 11745193 10.1002/1097-0142(20011115)92:10<2585::aid-cncr1611>3.0.co;2-#

[20] Ahmad SZ, Rajanbabu A, Vijaykumar DK, Haji AG, Pavithran K. A prospective comparison of perioperative morbidity in advanced epithelial ovarian cancer: Primary versus interval cytoreduction—experience from India. South Asian journal of cancer. 2015;4: 107–10. doi: 10.4103/2278-330X.173171 26942138 10.4103/2278-330X.173171 PMC4756482

[21] Greimel E, Kristensen GB, van der Burg ME, Coronado P, Rustin G, del Rio AS, et al Quality of life of advanced ovarian cancer patients in the randomized phase III study comparing primary debulking surgery versus neo-adjuvant chemotherapy. Gynecologic oncology. 2013;131: 437–44. doi: 10.1016/j.ygyno.2013.08.014 23994107 10.1016/j.ygyno.2013.08.014

[22] Onda T, Satoh T, Saito T, Kasamatsu T, Nakanishi T, Nakamura K, et al Comparison of treatment invasiveness between upfront debulking surgery versus interval debulking surgery following neoadjuvant chemotherapy for stage III/IV ovarian, tubal, and peritoneal cancers in a phase III randomised trial: Japan Clinical Oncology Group Study JCOG0602. European journal of cancer (Oxford, England: 1990). 2016;64: 22–31. doi: 10.1016/j.ejca.2016.05.017 27323348 10.1016/j.ejca.2016.05.017

[23] Dai-yuan M, Bang-xian T, Xian-fu L, Ye-qin Z, Hong-Wei C. A meta-analysis: neoadjuvant chemotherapy versus primary surgery in ovarian carcinoma FIGO stageIII and IV. World journal of surgical oncology. 2013;11: 267 doi: 10.1186/1477-7819-11-267 24112995 10.1186/1477-7819-11-267 PMC3852916

[24] Morrison J, Haldar K, Kehoe S, Lawrie TA. Chemotherapy versus surgery for initial treatment in advanced ovarian epithelial cancer. The Cochrane database of systematic reviews. 2012: Cd005343 doi: 10.1002/14651858.CD005343.pub3 22895947 10.1002/14651858.CD005343.pub3 PMC4050358

[25] Zeng LJ, Xiang CL, Gong YZ, Kuang Y, Lu FF, Yi SY, et al Neoadjuvant chemotherapy for Patients with advanced epithelial ovarian cancer: A Meta-Analysis. Scientific reports. 2016;6: 35914 doi: 10.1038/srep35914 27804983 10.1038/srep35914 PMC5090201

[26] Liberati A, Altman DG, Tetzlaff J, Mulrow C, Gotzsche PC, Ioannidis JP, et al The PRISMA statement for reporting systematic reviews and meta-analyses of studies that evaluate health care interventions: explanation and elaboration. Annals of internal medicine. 2009;151: W65–94. 19622512 10.7326/0003-4819-151-4-200908180-00136

[27] Higgins JP, Thompson SG, Deeks JJ, Altman DG. Measuring inconsistency in meta-analyses. BMJ (Clinical research ed). 2003;327: 557–60. doi: 10.1136/bmj.327.7414.557 12958120 10.1136/bmj.327.7414.557 PMC192859

[28] Higgins JP, Thompson SG. Quantifying heterogeneity in a meta-analysis. Statistics in medicine. 2002;21: 1539–58. doi: 10.1002/sim.1186 12111919 10.1002/sim.1186

[29] Peters JL, Sutton AJ, Jones DR, Abrams KR, Rushton L. Comparison of two methods to detect publication bias in meta-analysis. Jama. 2006;295: 676–80. doi: 10.1001/jama.295.6.676 16467236 10.1001/jama.295.6.676

[30] Mullan RJ, Flynn DN, Carlberg B, Tleyjeh IM, Kamath CC, LaBella ML, et al Systematic reviewers commonly contact study authors but do so with limited rigor. Journal of clinical epidemiology. 2009;62: 138–42. doi: 10.1016/j.jclinepi.2008.08.002 19013767 10.1016/j.jclinepi.2008.08.002

[31] Chern JY, Curtin JP. Appropriate Recommendations for Surgical Debulking in Stage IV Ovarian Cancer. Current treatment options in oncology. 2016;17: 1 doi: 10.1007/s11864-015-0380-2 26714493 10.1007/s11864-015-0380-2

[32] van Meurs HS, Tajik P, Hof MH, Vergote I, Kenter GG, Mol BW, et al Which patients benefit most from primary surgery or neoadjuvant chemotherapy in stage IIIC or IV ovarian cancer? An exploratory analysis of the European Organisation for Research and Treatment of Cancer 55971 randomised trial. European journal of cancer (Oxford, England: 1990). 2013;49: 3191–201. doi: 10.1016/j.ejca.2013.06.013 23850170 10.1016/j.ejca.2013.06.013

[33] Hacker NF, Rao A. Surgery for advanced epithelial ovarian cancer. Best practice & research Clinical obstetrics & gynaecology. 2016 doi: 10.1016/j.bpobgyn.2016.10.007 27884789 10.1016/j.bpobgyn.2016.10.007

[34] Gadducci A, Cosio S, Zizioli V, Notaro S, Tana R, Panattoni A, et al Patterns of Recurrence and Clinical Outcome of Patients With Stage IIIC to Stage IV Epithelial Ovarian Cancer in Complete Response After Primary Debulking Surgery Plus Chemotherapy or Neoadjuvant Chemotherapy Followed by Interval Debulking Surgery: An Italian Multicenter Retrospective Study. International journal of gynecological cancer: official journal of the International Gynecological Cancer Society. 2017;27: 28–36. doi: 10.1097/igc.0000000000000843 27870700 10.1097/IGC.0000000000000843

[35] Medina-Franco H, Cortes-Gonzalez R, Lambreton-Hinojosa F, Fimbres-Morales A, Vargas-Siordia JC. Neoadjuvant Chemotherapy Increases R0 Cytoreduction Rate But Does Not Improve Final Outcome in Advanced Epithelial Ovarian Cancer. Annals of surgical oncology. 2016 doi: 10.1245/s10434-016-5704-3 27995454 10.1245/s10434-016-5704-3

[36] Kessous R, Laskov I, Abitbol J, Bitharas J, Yasmeen A, Salvador S, et al Clinical outcome of neoadjuvant chemotherapy for advanced ovarian cancer. Gynecologic oncology. 2016 doi: 10.1016/j.ygyno.2016.12.017 28041690 10.1016/j.ygyno.2016.12.017

[37] Kumar L, Pramanik R, Kumar S, Bhatla N, Malik S. Neoadjuvant chemotherapy in gynaecological cancers—Implications for staging. Best practice & research Clinical obstetrics & gynaecology. 2015;29: 790–801. doi: 10.1016/j.bpobgyn.2015.02.008 25840650 10.1016/j.bpobgyn.2015.02.008

[38] Sioulas VD, Schiavone MB, Kadouri D, Zivanovic O, Roche KL, O'Cearbhaill R, et al Optimal primary management of bulky stage IIIC ovarian, fallopian tube and peritoneal carcinoma: Are the only options complete gross resection at primary debulking surgery or neoadjuvant chemotherapy? Gynecologic oncology. 2017;145: 15–20. doi: 10.1016/j.ygyno.2017.02.023 28238354 10.1016/j.ygyno.2017.02.023 PMC5386177

[39] Meyer LA, Cronin AM, Sun CC, Bixel K, Bookman MA, Cristea MC, et al Use and Effectiveness of Neoadjuvant Chemotherapy for Treatment of Ovarian Cancer. Journal of clinical oncology: official journal of the American Society of Clinical Oncology. 2016 doi: 10.1200/jco.2016.68.1239 27601552 10.1200/JCO.2016.68.1239 PMC5477982

[40] Vergote IB, Van Nieuwenhuysen E, Vanderstichele A. How to Select Neoadjuvant Chemotherapy or Primary Debulking Surgery in Patients With Stage IIIC or IV Ovarian Carcinoma. Journal of clinical oncology: official journal of the American Society of Clinical Oncology. 2016 doi: 10.1200/jco.2016.69.7458 27646940 10.1200/JCO.2016.69.7458

[41] Aletti GD, Dowdy SC, Podratz KC, Cliby WA. Analysis of factors impacting operability in stage IV ovarian cancer: rationale use of a triage system. Gynecologic oncology. 2007;105: 84–9. doi: 10.1016/j.ygyno.2006.10.055 17157903 10.1016/j.ygyno.2006.10.055

[42] da Costa Miranda V, de Souza Fede AB, Dos Anjos CH, da Silva JR, Sanchez FB, da Silva Bessa LR, et al Neoadjuvant chemotherapy with six cycles of carboplatin and paclitaxel in advanced ovarian cancer patients unsuitable for primary surgery: Safety and effectiveness. Gynecologic oncology. 2014;132: 287–91. doi: 10.1016/j.ygyno.2013.12.002 24333355 10.1016/j.ygyno.2013.12.002

[43] Sato S, Itamochi H. Neoadjuvant chemotherapy in advanced ovarian cancer: latest results and place in therapy. Therapeutic advances in medical oncology. 2014;6: 293–304. doi: 10.1177/1758834014544891 25364394 10.1177/1758834014544891 PMC4206650

[44] Hoskins WJ, Bundy BN, Thigpen JT, Omura GA. The influence of cytoreductive surgery on recurrence-free interval and survival in small-volume stage III epithelial ovarian cancer: a Gynecologic Oncology Group study. Gynecologic oncology. 1992;47: 159–66. 1468693 10.1016/0090-8258(92)90100-w

