# Expression of Concern: Neoadjuvant chemotherapy versus primary debulking surgery in advanced epithelial ovarian cancer: A meta-analysis of peri-operative outcome

**DOI:** 10.1371/journal.pone.0285545

**Published:** 2023-05-04

**Authors:** The PLOS ONE Editors

After this article was published, similarities were noted between this article [1] and submissions by other research groups which call into question the validity and provenance of the reported results. In light of these issues, the *PLOS ONE* Editors issue this Expression of Concern.

PLOS attempted to discuss the concerns with the authors, but the authors either did not respond or could not be reached through the email addresses on file with the journal.
