# Supplementary figures and images for: Neoadjuvant chemotherapy versus primary debulking surgery in advanced epithelial ovarian cancer: A meta-analysis of peri-operative outcome
Source: PLoS One. 2017 Oct 23;12(10):e0186725. doi: 10.1371/journal.pone.0186725 (PMC5653345; doi:10.1371/journal.pone.0186725)

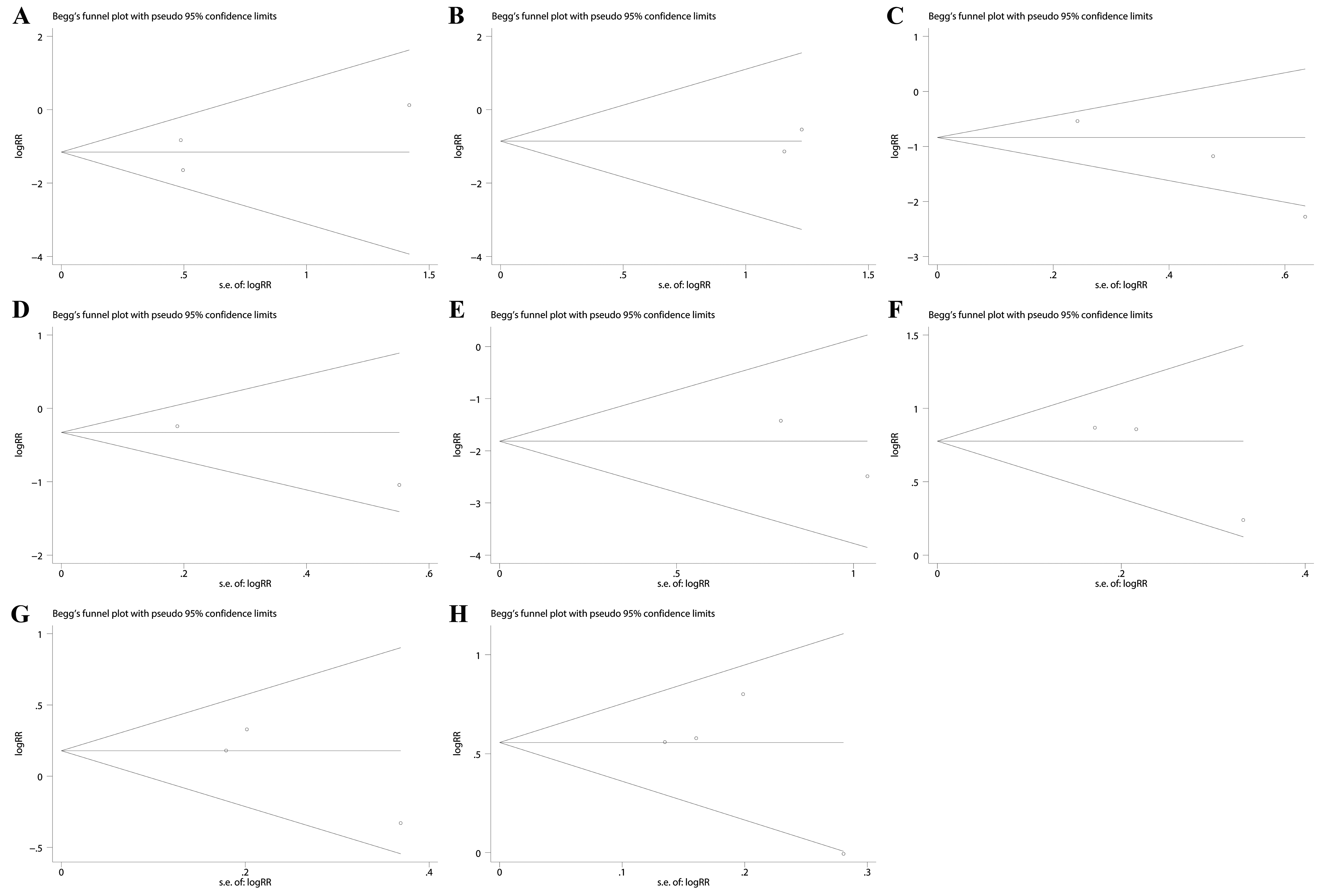

Supplement: S1 Fig — A, infection grade 3 or 4; B, gastrointestinal fistula; C, any grade 3 or 4 adverse event; D, patients transfusion; E, postsurgical death within 28 days; F, complete cytoreduction; G, residual disease 0–1 cm; H, optimal cytoreduction rate. RR = risk ratio, SE = standard error. (TIF) [file pone.0186725.s002.tif]
